# The accuracy of the MMSE in detecting cognitive impairment when administered by general practitioners: A prospective observational study

**DOI:** 10.1186/1471-2296-9-29

**Published:** 2008-05-13

**Authors:** Patrizio Pezzotti, Silvia Scalmana, Antonio Mastromattei, Domenico Di Lallo, the "Progetto Alzheimer" Working Group

**Affiliations:** 1 Lazio Sanità – Agenzia di Sanità Pubblica della Regione Lazio, Rome, Italy; 2 See "Acknowledgements"

## Abstract

**Background:**

The Mini-Mental State Examination (MMSE) has contributed to detecting cognitive impairment, yet few studies have evaluated its accuracy when used by general practitioners (GP) in an actual public-health setting.

**Objectives:**

We evaluated the accuracy of MMSE scores obtained by GPs by comparing them to scores obtained by Alzheimer's Evaluation Units (UVA).

**Methods:**

The study was observational in design and involved 59 voluntary GPs who, after having undergone training, administered the MMSE to patients with symptoms of cognitive disturbances. Individuals who scored ≤ 24 (adjusted by age and educational level) were referred to Alzheimer's Evaluation Units (UVA) for diagnosis (including the MMSE). UVAs were unblinded to the MMSE score of the GP. To measure interrater agreement, the weighted Kappa statistic was calculated. To evaluate factors associated with the magnitude of the difference between paired scores, a linear regression model was applied. To quantify the accuracy in discriminating no cognitive impairment from any cognitive impairment and from Alzheimer's disease (AD), the ROC curves (AUC) were calculated.

**Results:**

For the 317 patients, the mean score obtained by GPs was significantly lower (15.8 vs. 17.4 for the UVAs; p < 0.01). However, overall concordance was good (Kappa = 0.86). Only the diagnosis made by the UVA was associated with the difference between paired scores: the adjusted mean difference was 3.1 for no cognitive impairment and 3.8 for mild cognitive impairment. The AUC of the scores for GPs was 0.80 (95%CI: 0.75–0.86) for discriminating between no impairment and any impairment and 0.89 (95%CI: 0.84–0.94) for distinguishing patients with AD, though the UVA scores discriminated better.

**Conclusion:**

In a public-health setting involving patients with symptoms of cognitive disturbances, the MMSE used by the GPs was sufficiently accurate to detect patients with cognitive impairment, particularly those with dementia.

## Background

In industrialized countries, dementia is one of the main causes of death and disability for persons over 65 years of age. Dementing illnesses also have a strong impact on the quality of life of both patients and their families [1], requiring a complex organization of social and health services.

In Italy, among persons aged 65–84 years, the prevalence of any form of dementia has been estimated to be around 6%, whereas the estimated prevalence of Alzheimer's disease (AD) is around 2.5% [2]. When also considering persons older than 84 years, the estimated prevalence ranges from 5.9% to 6.4% for any dementia and from 3% to 3.3% for AD [3,4].

The initial stage of dementia is frequently not identified as such, with diagnosis often being performed two or three years after the first symptoms [5]. At present, it is not recommended that screening for non-symptomatic persons be performed, given the high rate of false-positive cases, whereas screening is instead recommended for symptomatic persons. In fact, early diagnosis is extremely important, given that there exists non-pharmacological and, to a lesser extent, pharmacological treatment capable of decreasing the severity of symptoms, retarding disease progression, and strengthening the residual cognitive abilities when neuronal impairment/death is not complete [6-11]. Furthermore, managing persons with dementia and, in particular, the progressive worsening of the disease entails implementing measures in the family environment and in the patient's general environment.

General practitioners (GP) could play a key role in detecting persons with symptoms that are suggestive of dementia (e.g., memory deficit and/or alterations in other cognitive processes). However, many of the neuropsychological scales and tests for predicting the onset of cognitive impairment are of limited use for GPs because of the time and complex training required to administer them [12-18].

The Mini-Mental State Examination (MMSE) is a widely used tool for assessing cognitive mental status which can be administered in less than 10 minutes by following simple instructions [19-23]. Though it cannot be used for making formal diagnoses [22], the MMSE has been used as a first step in detecting cognitive impairment [23-25]. However, few studies have evaluated its accuracy when used by GPs [24,25].

The objective of the present study was to determine in an actual public-health setting whether GPs can accurately detect a cognitive deficit using the MMSE in patients with suspected cognitive impairment. To this end, we compared the results of the MMSE applied by GPs to those of the MMSE applied by specialized neuropsychologists.

## Methods

The study was conducted as part of a the project "A Model for Estimating the Occurrence of Alzheimer's Disease for Creating a Regional Registry and Evaluating Healthcare Needs", conducted in Rome in 2004–2005 and financed by the Italian Ministry of Health. The project was performed in compliance with the Declaration of Helsinki [26] and was approved by the ethics committee of the "Fatebenefratelli Hospital" (Rome) in February 2004. Four specialized outpatient centres for dementia participated in the Project (referred to as "*Unità Valutativa Alzheimer*", UVA; Alzheimer's Evaluation Units) [27]. The objectives of the project were: 1) to define criteria for identifying persons with dementia in the general population; 2) to estimate the incidence and prevalence of dementia; 3) to construct an index of severity of dementia; 4) to identify neuropsychological, behavioural, and neurophysiological predictors of disease progression; 5) to quantify the resources used in relation to the disease; and 6) to provide useful information for the planning and development of an integrated model of service provision based on the network of available services.

In 2004–2005, we contacted the heads of the four Health Districts in which the above-mentioned UVAs were located, asking them to recruit GPs who had a private practice in their Health District. Eighty GPs volunteered to attend a meeting in which the study was presented; at this meeting, the GPs were first asked to participate in a course on dementia, particularly AD, with the aim of updating their knowledge on diagnostic criteria and patient management and to train them in the use of the MMSE, including the interpretation of the scores (for example, regarding questions on dates, if the answer was off by one day, then the GPs were instructed to consider this as a wrong answer).

Sixty-five GPs participated in the course. Of these, 59 agreed to participate in the MMSE evaluation. The GPs, based on their patients' records, were first asked to identify persons over 65 years of age who had already been diagnosed with any form of dementia and those with memory or cognitive disturbances that were causing difficulties in daily-living activities (reported by the patient him/herself or a family member) yet who had not suffered from pathologies other than dementia that cause cognitive impairment (e.g., Down Syndrome) The GPs were then asked to administer the MMSE (Italian version [28]), following the instructions for use, to those who had not already been diagnosed. All evaluated patients, or relatives, were asked to sign an informed consent form.

The total score for the MMSE ranges from 0 to 30; scores > 24 indicate basically no cognitive impairment; scores < 18 indicate severe cognitive impairment [20]. For persons with a score of 24 or lower (corrected by age and educational level using the score-adjustment coefficients proposed by Magni et al. [28]), the GPs used a standardized form to collect, with the help of patients' family members, demographic data, medical history (including pharmacological use), and family history of dementia. The GP then invited the patient to undergo a clinical examination at the UVA, which was scheduled within six months of the GP's evaluation. The clinical evaluation at the UVA was based on a standardized protocol and included cognitive and familial anamnesis, neurological examination, blood examination, neuroimaging (i.e., computed tomography or magnetic resonance), neuropsychological evaluation (i.e., MMSE, the Mental Deterioration Battery [14], the Stroop Test [29]), functional evaluation [i.e., Activities of Daily living (ADL) [30] and Instrumental Activities of Daily Living (IADL) [31]] and behavioural evaluation through the Neuropsychiatric Inventory (NPI) [32]. The diagnosis of any form of dementia was made according to DSM IV criteria [33]; the diagnosis of AD was made according to NINCS-ADRDA criteria [34]; that of vascular dementia according to NINDS-AIREN criteria [35] and that of frontotemporal dementia according to the Lund and Manchester criteria [36]. Mixed dementia was defined as the presence of both AD and vascular dementia. Mild cognitive impairment (MCI) was diagnosed according to Petersen et al. and Ritchie & Touchon criteria [37-40].

### Statistical analysis

Of the patients with suspected cognitive impairment identified by the GPs, we excluded from the analysis those being followed by an UVA and those who in the previous four years had been prescribed inhibitors of acetylcholinesterase (AChE), which are indicative of a diagnosis of dementia.

The scores of the MMSE administered by the GPs were compared to those of the MMSE administered by the UVAs. To examine agreement, the difference between the scores was plotted against their mean (Bland-Altman plot) [41]. The level of agreement was measured by the mean difference and the standard deviation of the differences. The precision of the estimated limits of the agreement was calculated assuming that differences basically followed a Normal distribution [41]. To provide a synthetic measurement of interrater agreement, the Kappa statistic was calculated using weights defined as follows:

weights = 1 - [(i - j)/(k - 1)]^2^

where i and j indicate the scores provided by the two raters and k is the maximum number of possible ratings (in this case 31 because the MMSE varies from 0 to 30). This measure of agreement is scaled to be 0 when the level of agreement is what would be observed by chance and 1 when there is perfect agreement.

To evaluate factors associated with the magnitude of the difference between scores, a linear regression model was used, considering this difference as a dependent variable and, as independent variables, age (categorized into three classes), gender, educational level (categorized into four classes), and the diagnosis made by the UVA (i.e., AD, other forms of dementia, MCI, and no cognitive impairment). The estimated standard errors were corrected for the clustering effect due to potential correlation among scores for the MMSE performed by the same GP [42].

To quantify the accuracy of the MMSE administered by the GP and by the UVA in discriminating unconfirmed cognitive impairment from any type of cognitive impairment (i.e., MCI, AD, and other types of dementia) and from AD, the ROC curves (AUC) were calculated. Finally, the sensitivity, specificity, the percentage of cases correctly classified, the positive predictive value (PPV), and the negative predictive value (NPV) were calculated for each score of the crude and adjusted MMSE administered by GPs and UVAs.

## Results

The GPs identified 552 individuals, of whom 155 had been previously diagnosed with dementia. Of the remaining 397 individuals, 28 were not tested; for 15 of these individuals, testing was not possible (e.g., they were institutionalized), whereas the remaining 13, though not tested, were nonetheless sent to the UVA based on evidence of cognitive impairment. Thus the GPs performed the MMSE on 369 individuals. Of these, 11 were excluded because the MMSE score exceeded 24, and 41 were excluded from the comparison because the MMSE was not performed by the UVA. Thus 317 individuals were included in the present analysis (82% of the eligible candidates).

Most of the 317 patients were women (73.5%); the most represented age group was that of persons 75–84 years of age (48.6%); and the largest proportion of participants had a low educational level (0–5 years of schooling) (68.1%). After the visit to the UVA, 84 (26.5%) of the cases with suspected cognitive impairment were not confirmed, and 40 (12.6%) were defined as affected by MCI; AD was the most frequent diagnosis of dementia (n = 95; 30.0%). Among the other types of dementia, there were 56 cases of mixed dementia, 33 cases of vascular dementia, 7 cases of frontotemporal dementia, and 2 undetermined cases. When considering both the cases identified with this study and those already diagnosed, the estimated prevalence was 3.5% for dementia (1.8% for AD) among persons aged 65 years or older who were followed by the 59 GPs.

**Figure 1:**
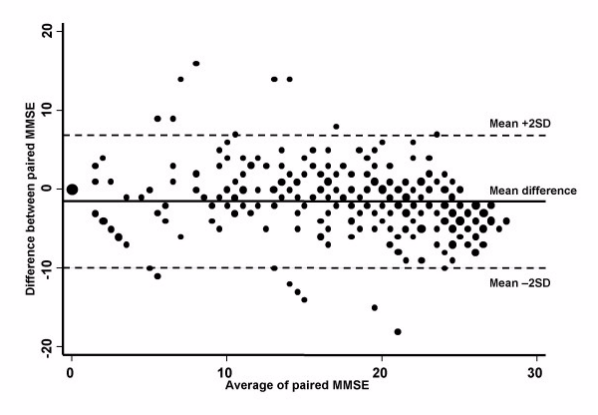
**Scatter plot of differences between paired MMSE scores and their average; Rome, Italy, 2005.** The diameter of the circle points are proportional to the frequency of the individuals with same difference and same average.

The mean crude score of the MMSE was 15.8 when the test was administered by the GPs [standard deviation (SD): 7.96, median: 18, range: 0–27, interquartile range (IQR): 11–22] and 17.4 when it was administered by the UVA (SD: 9.06, median:19, range: 0–30, IQR: 12–25). The median time between the two evaluations was 43 days (IQR: 17–119), during which deterioration in MMSE scores could be expected. The adjusted mean score (by educational level and age) was 16.0 when administered by the GPs [SD: 7.57, median: 18.5, range: 0.4–24.7, IQR: 12.1–22.0] and 17.7 when administered by the UVAs (SD: 8.74, median: 20.0, range: 0–30, IQR: 12.0–24.8). The mean and the median differences between paired scores (both crude and adjusted) were 1.58 (SD: 4.28) and 1 (IQR = -1; 4), respectively. Both the mean and the median differences were significantly different from zero (p < 0.01, by Student's t and Wilcoxon matched pair test). Figure 1 shows the difference between the paired measurements against their mean. Around 95% of the differences were between -10.0 and +6.8; for 29.4% of the participants, the difference between paired MMSE scores was 5 or more. The Kappa statistic was 0.86.

Table 1 shows the unadjusted and adjusted mean differences between paired scores obtained from the univariate and multivariate linear regression models, considering as independent variables age (categorized into three classes), gender, educational level (categorized into four classes), and the diagnosis made by the UVA (i.e., AD, other forms of dementia, MCI, unconfirmed cognitive impairment). At the univariate analysis, the mean differences were similar for males and females. The mean differences significantly varied by age class, with patients aged 65–74 years old having the largest mean differences, whereas those aged ≥85 years old had a mean difference very close to zero. For educational level, those with ≥ 14 years of schooling had the largest mean difference, although this was not statistically significant. For the UVA diagnosis, there was a significant difference among the 4 groups, with very large differences for those with no cognitive impairment and those with MCI. At the multivariate analysis, only the mean differences by type of diagnosis still varied significantly, showing that the age effect found at the univariate analysis was mainly attributable to confounding due to the type of diagnosis. We also explored other potential factors that could explain the difference between the paired MMSE scores. However, the UVA scores were, on average, higher in each participating UVA (data not shown). Furthermore, we did not find an association with the time between the two administrations. In fact, the mean differences were -2.8 for patients re-tested at the UVAs within 17 days, -1.0 for those re-tested between 19 and 42 days, -2.3 for those re-tested between 43 and 118 days, and -0.2 for those re-tested after 118 days.

**Table 1:** Crude and adjusted mean differences of paired MMSE scores obtained by GPs and UVAs for several patient characteristics; Rome, Italy, 2005

	Unadjusted mean difference	SE	p-value	Adjusted mean difference	SE	p-value
**Gender**			0.98			0.43
Female	-1.58	0.43		-1.72	0.37	
Male	-1.60	0.54		-1.21	0.49	
						
**Age (in years)**			0.02			0.11
65–74	-2.19	0.51		-1.41	0.44	
75–84	-2.09	0.43		-1.97	0.39	
≥ 85	-0.18	0.57		-1.05	0.55	
**Educational level (years of schooling)**			0.58			0.58
0–5	-1.49	0.45		-1.66	0.33	
6–8	-1.56	0.57		-1.09	0.58	
9–13	-1.45	0.63		-1.20	0.55	
≥ 14	-2.95	0.96		-2.56	1.31	
						
**Diagnosis made by UVA**			< 0.01			< 0.01
Unconfirmed cognitive impairment	-4.00	0.44		-3.92	0.54	
Mild cognitive impairment	-3.22	0.58		-3.25	0.59	
Alzheimer's disease	-0.17	0.58		-0.16	0.53	
Other types of dementia	-0.20	0.46		-0.29	0.48	
						
**Overall**	-1.58	0.37				

Note: Negative values in the mean differences indicate that the GPs provided higher scores than the UVAs. Adjusted mean differences are calculated from a multiple regression linear model, in which the independent covariates were all of the variables reported in the table. Standard errors of the estimated parameters of the model are corrected for clustering among patients evaluated by the same GP. P-values are adjusted Wald-tests evaluating the probability of having obtained these estimates under the null hypothesis that they were equal to zero (i.e., no difference among levels).

**Figure 2:**
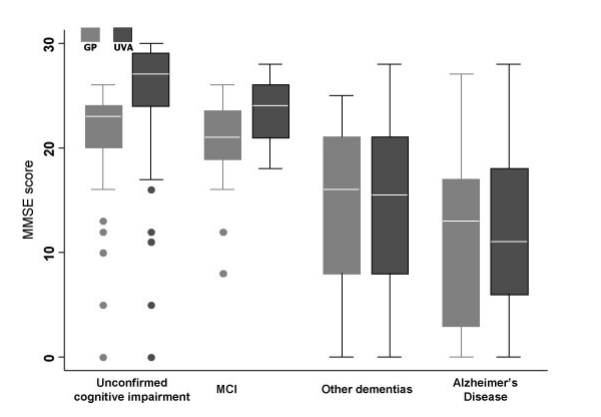
Box-plots of the MMSE scores obtained by GPs and UVAs, stratified by diagnosis made by UVAs; Rome, Italy 2005.

Figure 2 shows the box-plots of the MMSE scores, according to the GPs and UVAs, by type of UVA diagnosis. Overall, according to both GPs and UVAs, the scores were highest for persons with unconfirmed cognitive impairment, intermediate for those with MCI and lower for those with AD or other types of dementia. For persons with unconfirmed cognitive impairment or MCI, the UVAs, on average, gave higher scores than the GPs, whereas the scores were similar for persons with AD or other types of dementia. The results were very similar when adjusting the scores for age and educational level (data not shown in figure).

**Figure 3:**
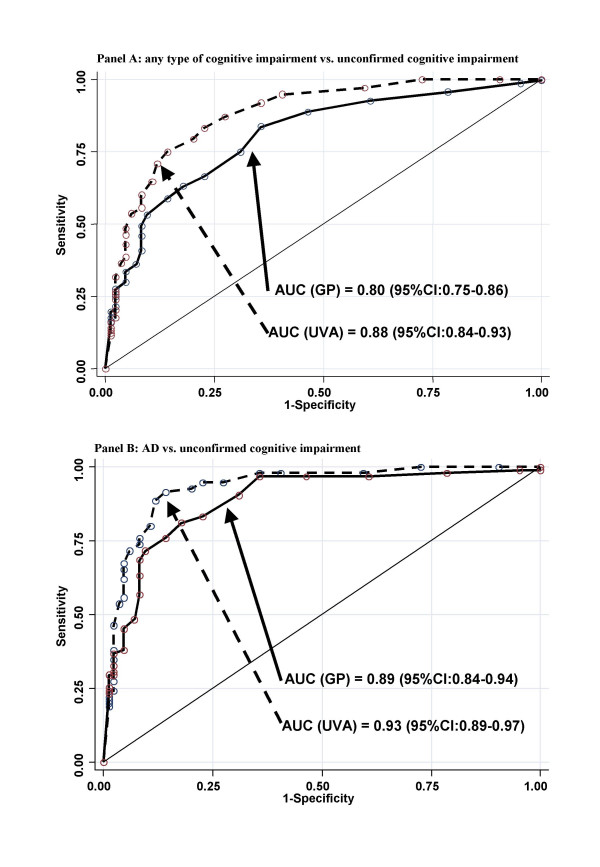
Accuracy of the MMSE scores obtained by GPs and UVAs in discriminating individuals with no cognitive impairment from those with cognitive impairment (panel A) and from those with Alzheimer's disease (panel B).

Figure 3 shows the graphs of the ROC curves of the crude MMSE scores according to the GPs and the UVAs, which illustrate the ability to discriminate unconfirmed cognitive impairment from any type of cognitive impairment (i.e., MCI, AD, and other types of dementia) (panel A) and from AD (panel B). In both cases, both the scores given by the GPs and those by the UVAs were able to discriminate (as shown by the AUC), though the scores given by the UVAs consistently provided a significantly better discrimination (p < 0.01). The results were very similar when adjusting the scores for age and educational level (data not shown in figure).

Finally, we calculated the sensitivity, the specificity, the percentage of correct classifications, the PPV, and the NPV for each crude MMSE score provided by GPs and by UVAs, which was used as cut-off to evaluate the accuracy in discriminating persons with and without cognitive impairment and with AD from those with unconfirmed cognitive impairment. Table 2 shows the results for cut-off values between 16 and 26. Regarding the cut-off value to better discriminate between persons with and without cognitive impairment, the value of 21 provided the highest percentage (i.e, 79.5%) of persons correctly classified when using the MMSE scores given by the GPs; when considering the MMSE scores given by the UVAs, the value that provided the highest percentage of persons correctly classified was 26 (i.e., 85.5%). Regarding the cut-off value to better discriminate persons with AD from those with no cognitive impairment, there was no single cut-off value for MMSE scores given by GPs. In fact, both the values 18 and 21 provided the highest percentage of persons correctly classified (i.e., 81.6%). The better cut-off value of the MMSE scores given by the UVAs was 21 (88.8% correctly classified).

**Table 2:** Sensitivity, specificity, and percentage of correct classification of individuals with and without cognitive impairment and those with AD and without cognitive impairment for different cut-off scores of the crude MMSE scores obtained by GPs and by UVAs; Rome, Italy, 2005

		**Crude MMSE obtained by GPs**					**Crude MMSE obtained by UVAs**				
	MMSE cut-off score	Sensitivity	Specificity	% correctly classified	PPV	NPV	Sensitivity	Specificity	% correctly classified	PPV	NPV

**Classification of individuals with and without cognitive impairment**	16	58.8%	85.7%	65.9%	92.0%	41.1%	53.7%	94.1%	64.4%	96.2%	42.3%
	17	63.1%	82.1%	68.1%	90.7%	42.9%	55.8%	91.7%	65.3%	94.9%	42.8%
	18	66.5%	77.4%	69.4%	89.1%	44.5%	60.1%	91.7%	68.5%	95.2%	45.3%
	19	75.1%	69.1%	73.5%	87.1%	45.5%	64.8%	89.3%	71.3%	94.4%	47.8%
	20	83.7%	64.3%	78.6%	86.7%	50.0%	70.8%	88.1%	75.4%	94.3%	52.1%
	21	88.8%	53.6%	79.5%	84.2%	58.7%	75.1%	85.7%	77.9%	93.6%	55.4%
	22	92.7%	39.3%	78.6%	80.9%	63.4%	79.4%	79.8%	79.5%	91.6%	58.3%
	23	95.7%	21.4%	76.0%	77.2%	66.0%	83.3%	77.4%	81.7%	91.1%	62.5%
	24	98.7%	4.8%	73.8%	74.2%	64.3%	87.1%	72.6%	83.3%	89.8%	67.0%
	25	99.6%	0.0%	73.2%	73.4%	57.1%	91.9%	64.3%	84.5%	87.7%	74.0%
	26	100.0%	0.0%	73.5%	73.5%	0.0%	94.9%	59.5%	85.5%	86.7%	80.7%

**Classification of individuals with AD and without cognitive impairment**	16	71.6%	90.5%	80.5%	89.5%	73.8%	71.6%	94.1%	82.1%	93.2%	74.5%
	17	75.8%	85.7%	80.5%	85.7%	75.8%	73.7%	91.7%	82.1%	90.9%	75.5%
	18	81.1%	82.1%	81.6%	83.7%	79.3%	75.8%	91.7%	83.2%	91.1%	77.0%
	19	83.2%	77.4%	80.5%	80.6%	80.2%	80.0%	89.3%	84.4%	89.4%	79.8%
	20	90.5%	69.1%	80.5%	76.8%	86.6%	88.4%	88.1%	88.3%	89.4%	87.1%
	21	96.8%	64.3%	81.6%	75.4%	94.7%	91.6%	85.7%	88.8%	87.9%	90.0%
	22	96.8%	53.6%	76.5%	70.2%	93.8%	92.6%	79.8%	86.6%	83.8%	90.5%
	23	96.8%	39.3%	69.8%	64.3%	91.7%	94.7%	77.4%	86.6%	82.6%	92.9%
	24	97.9%	21.4%	62.0%	58.5%	90.0%	94.7%	72.6%	84.4%	79.7%	92.4%
	25	99.0%	4.8%	54.8%	54.0%	80.0%	97.9%	64.3%	82.1%	75.6%	96.4%
	26	99.0%	0.0%	52.5%	52.8%	0.0%	97.9%	59.5%	79.9%	73.2%	96.2%

Note: Individuals whose score was equal to or lower than the reported cut-off value were classified as having cognitive impairment in the first section of the table and as having AD in the second section of the table.

## Discussion

The individuals with suspected cognitive impairment identified by GPs and sent to the UVAs had, on average, an MMSE score of 16, suggesting that a large proportion of them were in an advanced stage of disease without a specific diagnosis. Many of them were institutionalized or at home for other co-morbidities, and this study allowed them to be referred to specialized centres for dementia. Although dementia continues to be considered as a taboo for patients, families, and professionals in many countries, we observed that only 15 of the 397 individuals (3.8%) were not tested by GPs. This low percentage is likely the result of the advice and suggestions provided to the GPs during training to persuade patients and their families of the benefits of undergoing a specialized evaluation. It may also be due to the fact that the MMSE is easy to perform. Although there exist briefer tests (i.e., MIS, MISplus, MINIcog, GPCOG) which could be more attractive for use in general practice [43], we chose the MMSE because the others are not commonly used in Italian UVAs.

Our results showed that there was good agreement between the MMSE performed by GPs and that performed by neuropsycologists in the UVAs, although the scores obtained by the GPs were, on average, significantly lower. The overall concordance was good (Kappa statistic = 0.86), confirming the high test-retest reliability reported in several other studies [28]. Our results are also in agreement with those of a similar study performed in France, which found that the MMSE scores obtained by GPs were slightly yet significantly higher than those obtained by trained neuropsychologists. The concordance between paired scores in the French study was also similar to that in our analysis [25].

Age, gender, and educational level were apparently not associated with the difference between paired scores. There were significant differences when considering the diagnosis made by the UVA, specifically, for individuals with no cognitive impairment and those with MCI; however, it should be mentioned that the MMSE was not designed to detect patients with MCI. On average, the scores of the GPs decreased with increasing severity of dementia, with the highest median score for individuals with no cognitive impairment and the lowest median score for those with AD. A similar yet clearer trend was observed for the scores of the UVAs. It should be considered that the MMSE was always administered first by the GPs and then by the UVAs (after a median time of 43 days), during which time deterioration in MMSE scores could be expected; however, the time between assessments was not related to the degree of discrepancy. Other possible reasons for the difference between MMSE scores could be related to the site, the assessor and the time of administration, as well as to the specific items of the MMSE, yet no data were available to explore these hypotheses. We cannot exclude the possibility that for some patients with important symptoms, or because specifically requested by the patient's family, the GPs forced the MMSE to be 24 or less (i.e., the score needed to warrant a specialised visit by the UVA).

The scores of the GPs showed good accuracy in discriminating individuals with no cognitive impairment both from those with cognitive impairment (AUC = 0.80) and even more so from those with AD (AUC = 0.88). However, the scores of the UVAs discriminated significantly better than those of the GPs. Of note is the finding that, although the MMSE score has been shown to be influenced by age and educational level [22], we did not find a higher accuracy when adjusting the scores based on the Italian normative sample [28] for either the scores of the GPs or those of the UVAs. This was probably because of the relative homogeneity of the study population, which was characterized by a large percentage of persons with a low educational level (i.e., 0–5 years of education) and aged 75–84 years old.

The sensitivity and the specificity of several MMSE cut-off scores in discriminating individuals with no cognitive impairment from those with impairment and from those with AD differed from those reported in a previous study [24], in which analogous cut-offs showed lower sensitivity and higher specificity. However, these differences can probably be attributed to the fact that we did not include those individuals who were likely to have had no cognitive impairment (i.e, MMSE score of GP > 24), thus increasing the sensitivity and simultaneously reducing the specificity. Furthermore, we performed a cross-sectional evaluation to identify cases with cognitive impairment, whereas the previous study used the MMSE to predict AD after two years among persons initially not diagnosed with AD. We found that a cut-off between 18 and 21 provided the highest percentage of persons correctly classified. If the MMSE were to be used as a screening tool by GPs, a cut-off in this range should be considered.

In our study, all types of dementia other than AD, given the low numbers, were considered as "other types of dementia", yet we did not evaluate whether the MMSE scores of the GPs or the UVAs were capable of discriminating among the different types in this category. One of the objectives of our study was to identify persons with slightly compromised cognitive capabilities. We considered MCI as an intermediate state between normal aging and dementia [44] and which has been suggested to be a predictor of dementia [44,45], although it has been estimated that 11–40% of persons with MCI do not worsen over time and may revert to normal cognitive abilities [46]. To this regard, some authors have suggested that a non-pharmacological approach based on cognitive rehabilitation and a correct lifestyle may contribute to preventing MCI and dementia [47].

The MMSE scores of the GPs for individuals with no cognitive impairment were, on average, slightly higher than those for individuals with MCI, yet the difference was not statistically significant (p = 0.1); by contrast, the difference between the MMSE scores of the UVAs for these two groups was statistically significant (p < 0.01). This result seems to suggest that the MMSE, when applied by GPs, is of limited usefulness in distinguishing persons with MCI from those with no cognitive impairment. However, we have to take into account that in this study we included only those persons with scores ≤ 24 according to the GPs.

One of the strengths of our study is that we evaluated the reliability and accuracy of the MMSE in a sample of individuals who were not selected and who were in a true general-medicine setting. The major limitation of our study is that the GPs were asked to identify only those patients who they suspected to have a cognitive deficit (see selection criteria in the "Methods" section), and only those patients who scored 24 or less were evaluated by the UVAs. Thus we do not know how accurate the MMSE used by GPs would be in detecting cognitive impairment in patients not suspected to have a deficit (i.e., not sampled) or in patients with a suspected deficit yet with a score greater than 24. With regard to the latter patients, we feel that it is reasonable to assume that there were very few missed cases with cognitive impairment, as suggested by the results shown in Figure 2; furthermore, for patients who scored more than 24 yet whose symptoms were extremely serious, it was suggested that the GPs refer them to the UVA, and no cases of suspected cognitive impairment were confirmed. Another limit of our study is that the single items of the MMSE were not collected, so that we were not able to evaluate more in-depth whether or not disagreement between paired scores was due to specific aspects of the examination. Moreover, patients with borderline scores (23 or 24) according to the GPs had a greater probability of receiving higher UVA scores than patients with scores far below the cut off, simply because of measurement error and daily variation. In particular, we cannot exclude the possibility that some of the GPs may have assigned a score of 24 to persons who, in their opinion, based on clinical observation or information provided by family members, were affected by cognitive impairment. Another limit that should be taken into account is that the GPs were not randomly selected and they underwent a training session, limiting the representativeness with respect to the general community. Furthermore, the UVAs were not blinded to the GPs' MMSE scores, because patients were invited to go to the UVA based on the MMSE score. However, it is unlikely that the UVAs were conditioned by the score given by the GP. Finally, in this observational study, those who administered the MMSE in the UVAs were not blinded to the cognitive and familial anamnesis, the neurological and blood examinations, neuroimaging, and the neuropsychological, functional and behavioural evaluations. However, in the UVAs' normal clinical practice, the MMSE is administered and scored without taking into account the results of the other measurements for making the clinical diagnosis. Thus it is unlikely that this would have biased the results.

## Conclusion

In conclusion, our study shows that the MMSE can be used in a general-medicine setting as a useful tool for identifying cognitive impairment in individuals with memory or other cognitive impairment. This could contribute to increasing the early identification of persons with MCI or dementia and thus to increasing the timeliness of pharmacological and non-pharmacological treatment for delaying progression, as well as possible public-health measures for reducing the social impact for the affected individuals and their families.

## Competing interests

The authors declare that they have no competing interests.

## Authors' contributions

PP conceived the initial idea of the study, performed the statistical analysis, drafted and revised the manuscript. SS participated in the conception of the study project, supervised all the phases of the project (i.e., training, data collection, activities coordination, data registration), conceived the initial idea of the study, drafted and revised the manuscript. AM participated in the conception of the study project, supervised all the phases of the project (i.e., training, data collection, activities coordination, data registration), revised the manuscript. DDL participated in the conception of the study project, revised the manuscript and contributed especially to the intellectual content. All authors read and approved the final manuscript.

## Pre-publication history

The pre-publication history for this paper can be accessed here:


